# Methodological Variation Among Studies Evaluating Pain Processing in Tendinopathy: A Scoping Review

**DOI:** 10.3390/jcm13247592

**Published:** 2024-12-13

**Authors:** Dhinu Jayaseelan, Andrew Post, Josiah Sault, John Mischke

**Affiliations:** 1 Program in Physical Therapy, The George Washington University, Washington, DC 20006, USA; 2 Division of Rheumatology, Northwestern University, Chicago, IL 60611, USA; andrew.post@northwestern.edu; 3 Rehabilitation Services, University of Illinois Hospital and Health Sciences System, Chicago, IL 60611, USA; jsault2@uic.edu; 4 Department of Rehabilitation Science, Tufts University, Seattle, WA 98104, USA; jake.mischke@tufts.edu

**Keywords:** pain assessment, physical therapy, tendon, sensitization

## Abstract

**Background/Objectives:** Tendinopathy is a condition associated with pain and limited function. While upper and lower extremity tendinopathies may have different functional implications, there have been a number of reports supporting different patterns of dysfunction in pain processing and inhibition. The purpose of this scoping review was to examine the methods across studies examining pain processing in patients with upper and lower extremity tendinopathy. **Methods:** Five electronic databases (PubMed, Scopus, CINAHL, the Cochrane Library, and SPORTDiscus) and gray literature sources were searched from inception through 15 April 2024, using appropriate keywords and relevant synonyms. **Results:** In total, 3219 titles were retrieved from the searches, with 43 studies retained for final inclusion. Of the 43 studies, 22 were specific to upper extremity tendinopathies, 19 were specific to lower extremity tendinopathies, and 2 studies included mixed samples. Physical testing to detect nervous system sensitization was most commonly performed using pressure pain thresholds. Although infrequent, questionnaire instruments were used mostly to include the central sensitization inventory. Substantial variation was noted across studies in mode of testing and instruments used, while patient demographics and inclusion criteria were not clearly reported in many instances. Thirty-one studies (72%) reported nervous system sensitization or dysfunction in tendinopathy, while 13 (28%) did not. **Conclusions:** While the difference between pain processing in tendinopathy is likely multifactorial, the results of this review identified substantial variability in methodology used and reporting in tendon pain research. As inconsistency in evidence can limit clinical guidance, efforts to standardize tendinopathy pain research appear warranted.

## 1. Introduction

Tendinopathy is a common musculoskeletal diagnosis associated with pain and loss of function [1]. Epidemiological studies report high incidence and prevalence rates of tendinopathies in the upper [2,3] and lower extremities [4,5]. Despite substantial research related to best practices in managing tendinopathy, positive long-term outcomes are not consistently seen. With patellar tendinopathy, nearly one-third of athletes with the condition do not return to sports after six months [6], while one study reported 53% of athletes with the condition retired from sport completely [7]. In a large longitudinal study of soccer players, 27% of all Achilles tendinopathies were recurrent injuries, indicating an incomplete initial recovery [8]. Without treatment, greater trochanteric pain syndrome (GTPS) persisted in 45% of patients at 11-year follow-up and may contribute to secondary conditions such as hip osteoarthritis [9]. Nearly one-third of patients with lateral elbow tendinopathy [10,11] and half of patients with rotator cuff tendinopathy have persistent pain more than one year after initial onset despite intervention [12]. The lack of improvement and negative impact of tendon pain on long-term function warrants additional investigation into optimal evaluation and management of these conditions.

Localized tendon pain aggravated by activity and relieved with relative rest is consistent with a nociceptive mechanism for pain [13]. However, the central nervous system (CNS) demonstrates plasticity in pathological states, and sustained peripheral nociceptive input may lead to the development of altered peripheral and central pain processing [14]. This phenomenon, known as sensitization, is defined by the International Association for the Study of Pain (IASP) as “increased responsiveness of nociceptive neurons to their normal input and/or recruitment of a response to normally subthreshold inputs” [15] and could be an underlying mechanism for prolonging an individual’s pain experience [16]. While multiple input and processing mechanisms are involved in a patient’s pain experience, the identification of peripheral or central pain processing to inform the selection of interventions (e.g., education, electrical stimulation, exercise, manual therapy, pharmaceuticals, etc.) may be beneficial in improving an individual’s level of function [13,16]. Identification of pain processing dysfunction is particularly important with tendinopathy, as lateral elbow and shoulder studies found the presence of nervous system sensitization at baseline may be associated with poorer long-term outcomes [17,18,19,20]. Therefore, evaluating the predominant pain mechanism(s) involved in tendon pain may be warranted.

Previous reviews revealed an association between enhanced CNS sensitization as measured by quantitative sensory testing (QST) and upper extremity tendinopathies, whereas lower extremity tendinopathies may be predominantly peripheral nociceptive pain states [21,22]. While differing tendon characteristics may play a role in the conflicting results, it is also possible that variability of assessment methodologies between studies contributes to the inconsistency. Partly because of heterogeneity among studies, international experts saw the need to recently form consensus statements on the terminology, suggested reporting, and core health-related domains (among others) associated with tendinopathy research [23,24,25]. Inconsistencies in tendinopathy research study design, methods, modalities examined, and self-report instruments make synthesizing conclusions on altered pain processing mechanisms difficult. A lack of consistency in methodology and reporting also impairs the ability of researchers to perform meta-analyses or similar synthesized judgments to guide clinical practice. The evaluation of testing strategies and reporting for tendon pain research has not been critically evaluated and synthesized. Determining the degree of variation across studies evaluating tendon pain is an important step in closing current knowledge gaps.

A thorough examination of methodology, one of the primary indications for performing a scoping review [26], in tendinopathy research is warranted to understand the strength of current recommendations and guide research. Therefore, the primary purpose of this scoping review was to examine the variability of methodology across studies examining pain processing in patients with tendinopathy. Additionally, this project sought to identify gaps in available evidence to inform future tendinopathy pain studies.

## 2. Materials and Methods

This scoping review was registered on the Open Science Framework (OSF) website and is available online: https://doi.org/10.17605/OSF.IO/59FNS. Reporting followed guidance of the Preferred Reporting Items for Systematic Reviews and Meta-Analyses extension for scoping reviews (PRISMA-ScR).

### 2.1. Data Sources and Searches

A comprehensive and systematic computerized search of the electronic databases PubMed, Scopus, CINAHL, the Cochrane Library, and SPORTDiscus was conducted to identify articles relevant to the clinical question. All articles from database inception through 15 April 2024 were considered for inclusion. With the assistance of a research librarian, key search terms and various combinations of synonyms related to the concepts of tendinopathy and pain processing were entered into each database. Specific search strategies are presented in Supplementary File S1. The reference lists of eligible articles were manually examined for key review articles and additional relevant studies. Finally, clinical trial registries, Google, and the Open Grey database were scanned for pertinent work not captured otherwise.

### 2.2. Eligibility Criteria

Studies were considered in this review if they related to the clinical question and had a full-text report published in the English language in a peer-reviewed journal. For inclusion, the study population needed to have a clinical diagnosis of tendinopathy, and testing had to incorporate a subjective and/or objective clinical assessment tool for alterations in pain processing. Assessment strategies to detect peripheral and/or central sensitization and involved pain mechanisms (e.g., nociceptive, neuropathic, nociplastic) were included. Dysfunction in pain processing was dependent on the instrument used (i.e., reduced thermal or pain thresholds, depressed conditioned pain modulation [CPM], higher scores on pain questionnaires). Subacromial impingement and GTPS, conditions most commonly attributed to tendon pathology [27,28], were included if the study authors clarified no other condition was likely. Studies were excluded if they were published in abstract form only, were not in the English language or reasonably translated to English, participants did not have a clinical diagnosis of tendinopathy, or testing could not detect peripheral and/or central pain processing or inhibition. Study protocols, intervention studies, case reports with less than 10 participants, and animal studies were also excluded.

### 2.3. Study Selection

Two reviewers independently screened the titles and abstracts for eligibility through the web-based software platform Covidence using the criteria determined a priori. After the preliminary search of the above databases, any article that included clinical assessment of pain processing in tendinopathy was retained for further analysis. In cases where details of the study methods were unclear, the study’s corresponding author was contacted for additional information. Studies were retained for further analysis if both reviewers voted for inclusion. Amongst those articles in which disagreement occurred (i.e., one vote to include, one vote to exclude), a third author blinded to previous voting made the final decision for inclusion. After full-text articles were obtained, two reviewers independently evaluated the study for appropriateness. If a unanimous decision regarding inclusion was not obtained based on the two reviewers’ decisions, a third author blinded to previous voting was consulted for a final vote. In each case of disagreement at any stage, a majority (i.e., 2 reviewers to 1) vote guided the decision to include or exclude a study.

### 2.4. Data Extraction and Reporting

Data from studies were extracted to standardized electronic forms using Excel based on available information in the published article. Grouping of data was subclassified into 3 thematic areas after concept mapping was completed by the research team: (1) participant demographics and reporting, (2) physical testing used, and (3) self-reported or questionnaire-based testing used. For demographic data, the following information was extracted: the presence of clear inclusion and exclusion criteria, the diagnostic criteria used for tendinopathy, sample size, participant age, participant sex, participant body mass index (BMI), whether participants were taking medications, whether participants had a single site of pain, and for comparative studies, whether there were significant between-group differences. While more extensive data were retrieved from each study (Supplementary File S2), for purposes of describing results, frequencies were captured to more easily highlight comparisons between pain mechanism testing strategies for upper and lower extremity tendinopathy. The presence of nervous system sensitization or dysfunction, as determined by the study’s statement of results, was also noted based on testing modalities and study design.

### 2.5. Quality Assessment

Individual study quality was not assessed for this scoping review. Instead, this study focused on the relevance of variation in testing modalities and reporting characteristics in tendon pain or possible evidence gaps. For study quality and risk of bias for many of the included studies, readers are referred to previously published works [21,22].

## 3. Results

### 3.1. Selection of Studies

**Figure 1:**
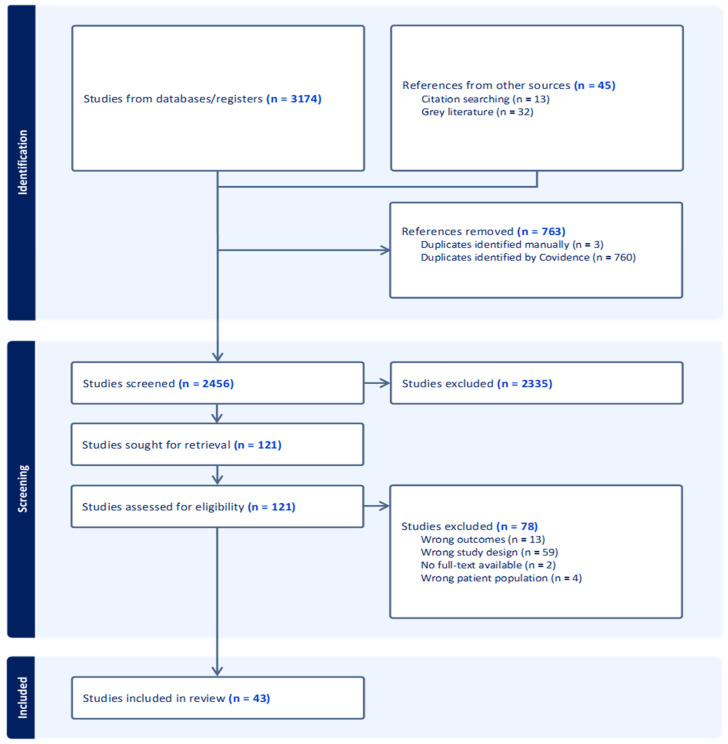
PRISMA flow diagram.

**Figure 2:**
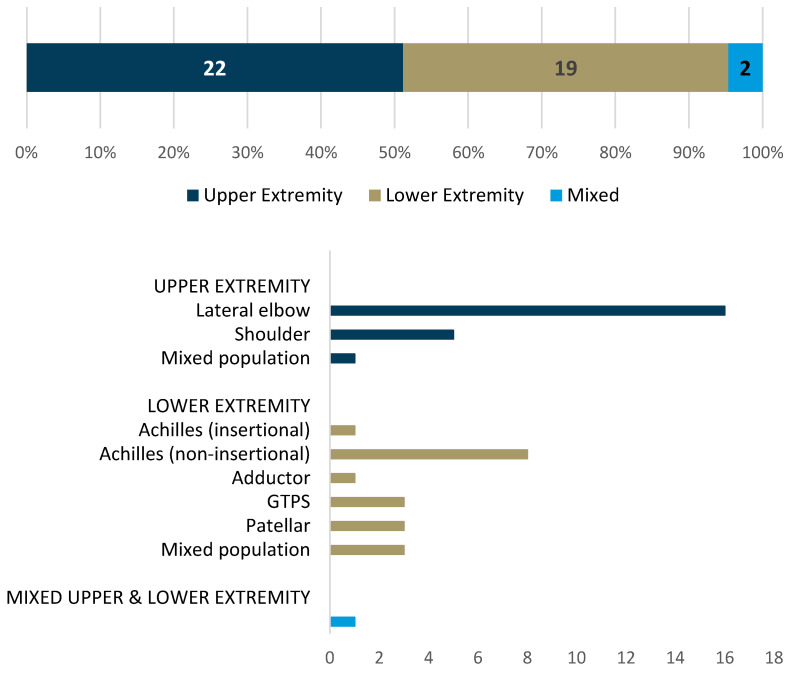
Number and proportion of included studies specific to each tendon.

In total, 3219 titles were retrieved from the searches. After the removal of duplicates, 2456 studies were screened, of which 43 studies were retained for final inclusion in this scoping review (Figure 1). Of the included studies, 32 (75%) used case–control designs [29,30,31,32,33,34,35,36,37,38,39,40,41,42,43,44,45,46,47,48,49,50,51,52,53,54,55,56,57,58,59,60], 9 (21%) used cross-sectional designs [61,62,63,64,65,66,67,68,69], 1 (2%) was a cohort study [70], and 1 (2%) was a post hoc analysis [71]. A breakdown of the number of studies by the specific tendon researched is presented in Figure 2. In total, included studies reported on 3728 participants, of which 2646 had a clinical diagnosis related to tendinopathy while 1082 participants were used for comparison.

### 3.2. Study Reporting of Demographics

Selected demographic data extraction variables were based on the ICON consensus statement [24] and items likely to affect pain perception (Table 1, File S2). Each of the studies (100%) clearly reported sample size, age, and sex of participants. The diagnostic criteria, inclusion criteria, and exclusion criteria were clearly reported in 95.3%, 88.4%, and 90.7% of studies, respectively. In comparative studies (n = 33), no significant between-group differences at baseline were reported by 18 studies (54.5%). BMI was calculated and reported in 53.5% of included studies. Importantly, while most studies stated recent corticosteroid injection was an exclusion criterion, only 30.2% of studies clearly stated that pain medication usage was not allowed by participants. Studies frequently excluded individuals with systematic pathology or chronic pain conditions (e.g., rheumatoid arthritis and fibromyalgia) and possible referred pain (e.g., cervical radiculopathy or surgery in the assessment of lateral elbow tendinopathy); however, no study (0%) explicitly noted that symptomatic participants had to have a single site of symptoms at the involved tendon to be included. No substantial differences were noted in demographic reporting between upper and lower extremity studies, except for BMI, which was reported by 40.9% and 68.4%, respectively.

### 3.3. Physical Examination Testing Modalities Used

**Figure 3:**
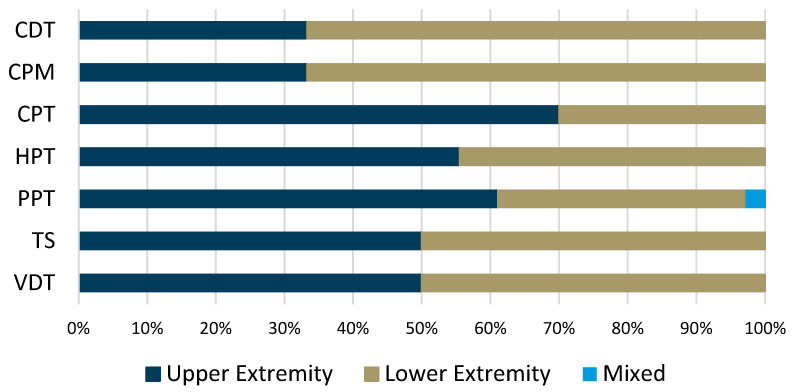
Proportion of studies using specific quantitative sensory testing instruments based on the type of tendon studied. Abbreviations: CDT—cold detection threshold; CPM—conditioned pain modulation; CPT—cold pressure threshold; HPT—heat pressure threshold; PPT—pressure pain threshold; TS—temporal summation; VDT—vibration detection threshold.

Of the 43 included studies, 39 (90.7%) used some form of physical testing measure to detect sensitization (Table 2). The proportion of physical testing modality use based on the tendon location can be seen in Figure 3. Four testing modalities were used by at least 20% of studies: pressure pain thresholds (PPT), cold pain thresholds (CPT), heat pain thresholds (HPT), and CPM. PPT were most commonly assessed, performed in 35 of the studies using physical testing (89.7%), and were employed in 22 upper extremity tendon studies compared to 13 lower extremity tendon studies. Twelve of the thirty-five studies (34.3%) examining PPT did not perform bilateral testing to include a remote site. Of the 12 studies, 8 examined upper extremity tendinopathies compared to 4 examining lower extremity tendinopathies. Five of the studies tested unilaterally and locally only (two upper extremity studies, three lower extremity studies), while seven studies tested bilaterally but not at a remote site (six upper extremity studies, one lower extremity study).

After PPT, CPT was the most commonly employed QST measure (10 studies). Testing for CPT was skewed towards upper extremity studies (n = 7, 70%), while only three lower extremity studies (30%) completed this assessment. Of the 10 studies, 3 (one upper extremity, two lower extremity) did not complete a bilateral assessment: 1 (upper extremity) was local only, 1 (lower extremity) was local and a remote elbow site, and 1 (lower extremity) was not clearly reported. HPT was assessed in nine studies (five upper extremity and four lower extremity). Similarly to CPT, some inconsistency was noted with unilateral versus bilateral testing. Three of the four (75%) lower extremity studies did not report a bilateral assessment, while five of the five upper extremity studies did report.

CPM was examined in nine studies. There was a skew towards being more used in lower extremity studies (n = 6, 66.7%) as compared to upper extremity studies (n = 3, 33.3%). Similar methodology and clear reporting were noted in studies using CPM.

Nervous system sensitization was detected using physical testing instruments in approximately 72% of all studies, but less frequently in the lower extremity (62.5% of the time) compared to the upper extremity (90%). When considering PPT, taken locally and/or at a remote site, 18 of 22 studies (81.2%) examining upper extremity tendons found sensitization (i.e., reduced thresholds compared to comparison groups), whereas 9 of 13 studies (69.2%) examining lower extremity tendons found sensitization. Of the 10 studies using CPT, 7 found evidence of sensitization, which included 5 of the 7 (71.4%) upper extremity studies and 2 of the 3 (66.7%) lower extremity studies. Similarly, sensitization was identified in four of five of the upper extremity studies and three of four of the lower extremity studies incorporating HPT. Dysfunction was found in six of nine (66.7%) studies using CPM; two of three (66.7%) were upper extremity studies, and four of six (66.7%) were lower extremity studies.

### 3.4. Self-Reported Instruments and Questionnaires Used

**Figure 4:**
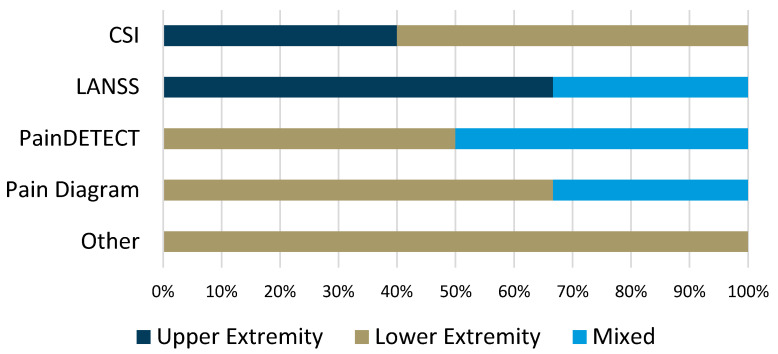
Proportion of studies using self-reported outcome or questionnaire instruments according to the type of tendon studied. Abbreviations: CSI—central sensitization inventory; LANSS—Leeds assessment of neuropathic pain symptoms and signs.

Of the included studies, a relatively small proportion (n = 11, 25.6%) used self-reported outcomes or questionnaires to detect nervous system sensitization. The most commonly used instrument was the central sensitization inventory (CSI), which was used in five studies, two (40%) examining upper extremity tendons and three (60%) examining lower extremity tendons (Table 3). The proportion of studies using questionnaires or self-reports by tendon location is presented in Figure 4. More lower extremity tendon studies (n = 7, 63.6%) compared to upper extremity tendons (n = 3, 27.3%) used questionnaires, while one study (9.1%) had a mixed population. Dysfunction was detected in seven (63.6%) of the studies using questionnaires, which often employed a cross-sectional design.

### 3.5. Presence of Nervous System Sensitization or Dysfunction

**Figure 5:**
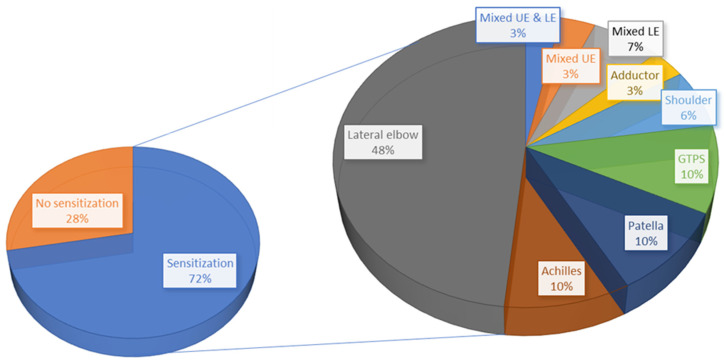
Percentage of studies detecting sensitization (**left**) including a breakdown of proportional tendon-specific contributions (**right**).

**Figure 6:**
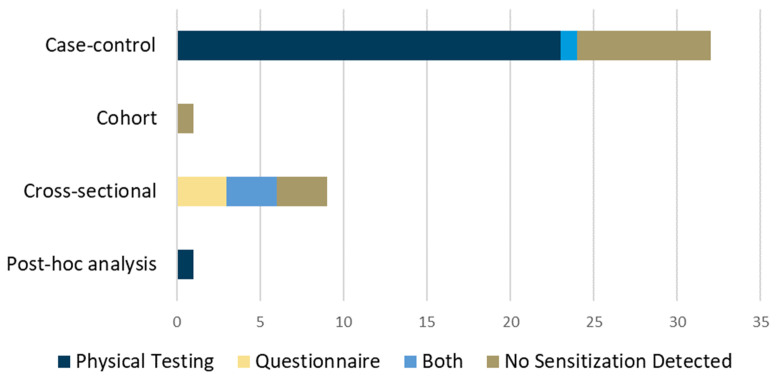
Number of studies, by design, detecting sensitization in tendon pain and the type of instrument used.

A primary intent of the included articles was to evaluate pain processing and mechanisms in persons with tendinopathy. Of the 43 included studies, 72% of studies (n = 31) detected peripheral and/or central sensitization in some capacity, while 28% (n = 12) did not. A tendon-specific breakdown of the studies detecting sensitization is presented in Figure 5. Lateral elbow tendinopathy was the most commonly studied tendon and most frequently associated with nervous system sensitization. The presence of sensitization in tendinopathy was also categorized by the study design (Figure 6).

## 4. Discussion

This scoping review sought to examine the methodological variation among studies examining pain mechanisms in tendinopathy. Based on results of the review, PPT is the most used testing modality to detect the presence of sensitization in individual with tendinopathy, questionnaire instruments were infrequently used, and participant reporting is an area requiring more specificity. Additionally, the variation in testing strategies and methodology may play a role in the conflicting results related to pain processing in patients with tendinopathy.

Our review found substantial procedural variation and a lack of clear reporting of QST protocols found across studies. Methodological rigor with the inclusion of matched controls for major demographics (e.g., age, sex, BMI) is essential to understand the sample studied, but particularly for comparing groups. Almost half of the studies comparing groups at baseline either had significant differences or did not report differences between groups. While significant between-group differences may be more relevant if studies are adequately powered for such analysis, comparing groups with different baseline characteristics makes comparison of conclusions impractical. Similarly to previous trials related to exercise for tendinopathy [72], inconsistent participant characteristic reporting was found in studies examining pain processing in tendinopathy. Only 53.5% of the studies included in this review calculated and reported BMI. Incomplete or missing participant demographics, including BMI, can limit interpretation and comparison to other studies. For example, multiple studies have identified a correlation between adiposity and pain [73,74], with one study identifying obese individuals to have more pressure pain sensitivity as compared to thermal sensitivity, indicating different testing modalities may have different results for different body morphologies [75]. Few studies (30.2%) explicitly stated that pain medication usage was not allowed. If participants were taking pain medication, their ability to perceive painful stimuli would be altered, seemingly invalidating the ability to detect differences between persons with or without a condition. Similarly, no study explicitly noted that participants with tendinopathy were excluded if they had any symptoms outside of the specific tendon of interest. While studies attempted to reduce the possibility of referred pain from the spine, studies did not clarify that participants were free of pain reports in any part of the body. This can challenge the ability to differentiate peripheral nociceptive pain from neuropathic from centrally mediated pain, particularly if other pain sites are not recorded and reported. Multi-site pain is a common complaint in persons with musculoskeletal pain and can be more common than single-site reports for many with chronic pain [76,77]. Without recognition of whether participants had pain in other areas, accurate comparisons between individuals with single-site pain or no pain become difficult.

In our review, upper extremity tendinopathies demonstrated high rates of the presence of centralized pain through QST compared to lower extremity tendinopathies, which aligns with previous studies that have compared these diagnoses [21,22]. Hypotheses for discrepancies within findings have previously highlighted differences in the unique functional demands of upper and lower extremity tendons, making direct comparison difficult [78]. While classically referred to for their recreational connotations of ‘tennis elbow’ or ‘golfer’s elbow’, elbow tendinopathy is often related to several occupations [79,80]. Musculoskeletal pain can contribute to substantial burden as it relates to vocational activities, including a considerable psychological impact [81]. Manual jobs and high physical strain at work also carry a negative prognostic relationship with lateral elbow and rotator cuff tendinopathy [82,83]. Alternatively, while patellar and Achilles tendinopathies can occur in the general population, they are frequently seen in athletes and recreationally active individuals [84,85]. Importantly, pain processing in athletes may be different than in non-athletes. For recreationally active individuals, and high-level athletes in particular, pain thresholds are often higher [86,87], and regular exercise can have a protective effect against the centralization of pain [88]. The negative impact of upper and lower extremity tendinopathy on function may contribute to the higher proportion of nervous system sensitization in the upper versus lower extremity seen in this and previous reviews [21,22].

In the studies reviewed, PPT was the most common physical testing modality used across studies. When comparing PPT of upper and lower extremity tendons, the administration of testing should be considered. Reliability and accuracy of testing require consistent testing positions. When testing PPT for tendinopathy, testing is typically conducted at the site of maximal pain. Using the Achilles tendon as an example, while it is perhaps performed but rarely reported, consistent angles of ankle dorsiflexion would be required to ensure appropriate and comparable tendon deformation given a consistent rate of application. Alternatively, a consistent resting position of elbow flexion is more easily achieved to allow exposure to the common extensor tendon, making elbow testing seemingly easier than the ankle. While a direct comparison should be considered with caution, the reliability of pressure algometry may be better for testing lateral elbow versus patellar tendinopathy [89,90]. A lack of standardized testing positions for different tendons likely contributes to the variability seen among the results of the pain assessment studies. The lack of consistency across studies seen in this review (e.g., lack of remote site algometry assessment, inconsistent bilateral thermal assessment) makes comparisons between upper and lower extremity tendinopathy sensitization challenging. Also, if a local site is solely tested, the presence of secondary hyperalgesia becomes difficult to detect, limiting the ability to accurately differentiate peripheral versus central sensitization [14]. It is also important to recognize different QST measures assess unique characteristics of pain processing and inhibition. For example, CPM assesses endogenous pain inhibition, TS can identify enhanced facilitation, while thermal and PPT findings may represent peripheral, central, or a mixed presentation of sensitization. However, normative QST data for the tendinopathy population does not yet exist, which would seemingly make standard and consistent testing essential. Recognizing the purpose of each tool and understanding the available evidence for QST findings in the tendinopathy population is helpful in applying appropriate clinical assessment and management strategies.

Questionnaires have been developed with the proposed purpose of identifying neuropathic and centrally mediated pain syndromes. These questionnaires can be useful to identify phenotypic profiles in a large sample of individuals and do so rapidly with low investigator and participant burden [91]. However, the psychometric properties of commonly used questionnaires have been called into question [92], and in some cases may represent psychological constructs more so than nociceptive function [93]. Also, while researchers may have provided specific instructions to participants completing the questionnaires, the instruments have not been previously validated in the tendinopathy population, making their utility unclear. This review found that when utilized, questionnaires were typically a part of cross-sectional study designs. Sensitization was detected with questionnaires in two-thirds of the cross-sectional studies, as compared to one of the case–control designs. Without a comparison sample of persons without the condition, or even persons with the condition and multiple pain sites, using these instruments with reportedly questionable psychometric properties warrants additional consideration.

Previous studies have examined pain mechanisms involved in tendinopathy [21,22]. This scoping review is unique to previous studies in that the methodological approaches across studies evaluating pain mechanisms in tendinopathy have not been examined. As noted within this review, participant characteristics were not clearly identified to appropriately determine their baseline and ability to report pain (e.g., resting intensity, pain sites, medication usage). Similarly, the mode of testing should be examined as it relates to pain mechanism conclusions. While PPT was commonly used, it may be a less effective modality in some populations, and tendon pain may be reported differently depending on the assessment used and tendon tested. While contemporary pain science research has positively impacted the quality of care delivered to individuals with musculoskeletal pain, much remains unknown. Physiologic and psychologic changes may create a pain mechanistic network or signature of sorts, particularly in the chronic pain population [94]. Identifying the variables and mechanisms associated with a pain condition can be useful in selecting appropriate treatment strategies. Using manual therapy as an example, there is moderate-quality evidence supporting effectiveness in improving mechanical hyperalgesia, whereas low-quality evidence supports its use for improving temporal summation or CPM [95]. These findings suggest certain interventions may have an effect on a specific aspect of pain, and pain detection strategies may need to change based on the mechanism involved. As additional findings regarding pain processing and inhibition in tendinopathy are presented, clinicians may be able to provide more precise management approaches.

Despite its novelty and potential benefit in guiding future research, this scoping review does carry a number of limitations. With all systematic, scoping, and narrative reviews, it is possible relevant works were not retained for inclusion. The search was conducted with a research librarian, using multiple electronic databases and a rigorous blinded screening process; however, recommendations and synthesized results may change with additional studies. Some may question the absence of study quality assessment. The primary focus of this review was to examine the variability of methods across studies, and therefore quality assessment of the included studies was not undertaken. Additionally, while variation in methodology and reporting may affect outcomes and conclusions, no explanatory research analysis was performed as part of this study. The quantitative impact of methodology on tendinopathy pain research is unclear.

Given the suboptimal outcomes in up to half of the individuals with tendinopathy [7,12], more research is needed. This scoping review highlights key areas of opportunity for developing stronger research methodology related to tendon pain. Differences across studies based on testing modality, testing methodology, and participant demographics may influence the conclusions drawn and capacity to make comparative statements across upper and lower extremity tendinopathies. Future study design and methodology should be guided by ICON consensus statements and reported thoroughly for reproducibility. Exploration of methodological standardization and instrument validation in the tendinopathy population should be considered. Studies using QST could follow previously established protocols [96]. Ideally, samples should be systematically and specifically described, adequately powered, and data appropriately analyzed. Statistically significant differences should be scrutinized for biologic and clinical relevance. In doing so, accurate conclusions can be made related to the predominant pain mechanism(s) involved in various tendinopathies, in turn allowing for more thorough understanding and targeted intervention strategies. With improved methodology in tendon pain research, clinicians can more confidently use evidence conclusions to effectively manage patients with tendinopathy, a condition often associated with protracted and incomplete recovery. If clear conclusions made from consistent methodology identify impairments or dysfunction (or lack thereof) in pain processing, treatment may be inclusive of interventions to address the appropriate mechanisms and pathways.

## 5. Conclusions

Musculoskeletal pain, including tendinopathy, is complex and multifactorial. Numerous instruments have been used to detect pain processing mechanisms clinically and in research settings. Tendon pain, while typically presenting as a focal location of symptoms, can be associated with altered central pain processing potentially contributing to poor long-term outcomes. Based on this scoping review, there appears to be variance in the methodology used to detect pain processing in studies evaluating upper and lower extremity tendinopathies. Consistent and thorough participant reporting and standardized procedures may minimize methodological variation to allow researchers and clinicians to draw appropriate conclusions across studies.

## Figures and Tables

**Table 1:** Number of studies clearly reporting relevant demographic information, n (%).

Tendon Studied	Inclusion Criteria	Exclusion Criteria	Diagnostic Criteria	Sample Size	Age	Gender	BMI	Medication Usage	Single Pain Site	No Between-Group Differences *
Total studies (n = 43)	38 (88.4%)	39 (90.7%)	41 (95.3%)	43 (100%)	43 (100%)	43 (100%)	23 (53.5%)	13 (30.2%)	0 (0%)	18/33 (54.5%)
UE studies (n = 22)	20 (90.9%)	21 (95.5%)	20 (90.9%)	22 (100%)	22 (100%)	22 (100%)	9 (40.9%)	6 (27.3%)	0 (0%)	10/19 (52.6%)
Lateral elbow (n = 16)	15 (94.8%)	16 (100%)	15 (94.8%)	16 (100%)	16 (100%)	16 (100%)	7 (43.8%)	3 (18.8%)	0 (0%)	8/14 (57.1%)
Shoulder (n = 5)	5 (100%)	5 (100%)	4 (80%)	5 (100%)	5 (100%)	5 (100%)	1 (20%)	3 (60%)	0 (0%)	2/5 (40%)
Mixed sample (n = 1)	0 (0%)	0 (0%)	1 (100%)	1 (100%)	1 (100%)	1 (100%)	1 (100%)	0/1 (0%)	0 (0%)	NA
LE studies (n = 19)	17 (89.5%)	17 (89.5%)	19 (100%)	19 (100%)	19 (100%)	19 (100%)	13 (68.4%)	7 (36.8%)	0 (0%)	7/13 (53.8%)
Achilles (n = 9)	9 (100%)	9 (100%)	9 (100%)	9 (100%)	9 (100%)	9 (100%)	6 (66.7%)	2 (22.2%)	0 (0%)	3/6 (50%)
Adductor (n = 1)	1 (100%)	1 (100%)	1 (100%)	1 (100%)	1 (100%)	1 (100%)	0 (0%)	1 (100%)	0 (0%)	0/1 (0%)
GTPS (n = 3)	3 (100%)	3 (100%)	3 (100%)	3 (100%)	3 (100%)	3 (100%)	3 (100%)	0 (0%)	0 (0%)	1/2 (50%)
Patellar (n = 3)	3 (100%)	3 (100%)	3 (100%)	3 (100%)	3 (100%)	3 (100%)	3 (100%)	3 (100%)	0 (0%)	2/3 (66.7%)
Mixed sample (n = 3)	1 (33.3%)	1 (33.3%)	3 (100%)	3 (100%)	3 (100%)	3 (100%)	1 (33.3%)	1 (33.3%)	0 (0%)	1/1 (100%)
Mixed UE/LE studies (n = 2)	2 (100%)	2 (100%)	2 (100%)	2 (100%)	1 (50%)	0 (0%)	0 (0%)	1 (100%)	2 (100%)	1/1 (100%)

Abbreviations: BMI—body mass index; GTPS—greater trochanteric pain syndrome; LE—lower extremity; UE—upper extremity. * Indicates studies that specifically reported no between-group differences; does not include studies without a comparison group.

**Table 2:** Number of studies utilizing specific physical examination assessments of pain, n (%).

Tendon Studied	Dysfunction Detected	CDT	CPM	CPT	HPT	PPT	TS	VDT
Total studies (n = 39)	28 (71.9%)	4 (10.3%)	9 (23.1%)	10 (25.6%)	9 (23.1%)	35 (89.7%)	6 (15.4%)	4 (10.3%)
Upper extremity studies (n = 22)	18 (90%)	2 (9.1%)	3 (13.6%)	7 (31.8%)	5 (22.7%)	22 (100%)	3 (13.6%)	2 (9.1%)
Lateral elbow (n = 16)	15 (93.8%)	1 (6.3%)	2 (12.5%)	5 (31.3%)	4 (25%)	16 (100%)	2 (12.5%)	2 (12.5%)
Shoulder (n = 5)	2 (40%)	1 (20%)	1 (20%)	1 (20%)	1 (20%)	5 (100%)	1 (20%)	0 (0%)
Mixed sample (n = 1)	1 (100%)	0 (0%)	0 (0%)	1 (100%)	0 (0%)	1 (100%)	0 (0%)	0 (0%)
Lower extremity studies (n = 16)	10 (62.5%)	2 (12.5%)	6 (37.5%)	3 (18.8%)	4 (25%)	13 (81.3%)	3 (18.8%)	2 (12.5%)
Achilles (n = 8)	3 (37.5%)	0 (0%)	4 (50%)	0 (0%)	1 (12.5%)	5 (62.5%)	1 (12.5%)	0 (0%)
Adductor (n = 1)	1 (100%)	0 (0%)	0 (0%)	0 (0%)	0 (0%)	1 (100%)	0 (0%)	0 (0%)
GTPS (n = 3)	3 (100%)	0 (0%)	2 (66.7%)	1 (33.3%)	1 (33.3%)	3 (100%)	2 (66.7%)	0 (0%)
Patellar (n = 3)	3 (100%)	1 (33.3%)	0 (0%)	1 (33.3%)	1 (33.3%)	3 (100%)	0 (0%)	1 (33.3%)
Mixed sample (n = 1)	0 (0%)	1 (100%)	0 (0%)	1 (100%)	1 (100%)	1 (100%)	0 (0%)	1 (100%)
Mixed studies (n = 1)	0 (0%)	0 (0%)	0 (0%)	0 (0%)	0 (0%)	1 (100%)	0 (0%)	0 (0%)

Abbreviations: CDT—cold detection threshold; CPM—conditioned pain modulation; CPT—cold pressure threshold; GTPS—greater trochanteric pain syndrome; HPT—heat pressure threshold; PPT—pressure pain threshold; TS—temporal summation; VDT—vibration detection threshold.

**Table 3:** Number of studies utilizing patient-reported questionnaire assessments of pain, n (%).

Tendon Studied	Dysfunction Detected	CSI	LANSS	PainDETECT	SSS	WPI	Pain Diagram	Other
Total studies (n = 11)	7 (63.6%)	5 (45.5%)	3 (27.2%)	2 (18.2%)	0 (0%)	0 (0%)	3 (27.2%)	1 (9.1%)
Upper extremity studies (n = 3)	2 (66.7%)	2 (66.7%)	2 (66.7%)	0 (0%	2 (66.7%)	2 (66.7%)	1 (33.3%)	0 (0%)
Lateral elbow (n = 2)	1 (50%)	2 (100%)	2 (100%)	0 (0%)	2 (100%)	2 (100%)	0 (0%)	0 (0%)
Shoulder (n = 0)	NA	NA	NA	NA	NA	NA	NA	NA
Mixed sample (n = 1)	1 (100%)	0 (0%)	0 (0%)	0 (0%)	0 (0%)	0 (0%)	1 (100%)	0 (0%)
Lower extremity studies (n = 7)	4 (57.1%)	3 (42.9%)	0 (0%)	2 (28.6%)	0 (0%)	0 (0%)	2 (28.6%)	1 (14.3%)
Achilles (n = 1)	0 (0%)	0 (0%)	0 (0%)	1 (100%)	0 (0%)	0 (0%)	1 (100%)	0 (0%)
Adductor (n = 0)	NA	NA	NA	NA	NA	NA	0 (0%)	NA
GTPS (n = 2)	2 (100%)	1 (100%)	0 (0%)	0 (0%)	0 (0%)	0 (0%)	1 (50%)	0 (0%)
Patellar (n = 2)	0 (0%)	1 (50%)	0 (0%)	0 (0%)	0 (0%)	0 (0%)	0 (0%)	1 (50%)
Mixed sample (n = 2)	2 (100%)	1 (50%)	0 (0%)	1 (50%)	0 (0%)	0 (0%)	0 (0%)	0 (0%)
Mixed studies (n = 1)	1 (100%)	0 (0%)	1 (100%)	0 (0%)	0 (0%)	0 (0%)	0 (0%)	0 (0%)

Abbreviations: CSI—central sensitization inventory; GTPS—greater trochanteric pain syndrome; LANSS—Leeds assessment of neuropathic pain symptoms and signs; SSS—symptom severity scale; WPI—widespread pain index.

## Data Availability

All relevant data are provided within the manuscript and in the Supplementary Materials.

## Supplementary Materials

The following supporting information can be downloaded at: https://www.mdpi.com/article/10.3390/jcm13247592/s1, File S1: Search String and Results; File S2: Data Extraction.
