# Supplementary material for: Methodological Variation Among Studies Evaluating Pain Processing in Tendinopathy: A Scoping Review
Source: J Clin Med. 2024 Dec 13;13(24):7592. doi: 10.3390/jcm13247592 (PMC11677147; doi:10.3390/jcm13247592)
Supplement: Supplementary file 1 [file jcm-13-07592-s001.zip › File S1 - Search String and Results - Tendinopathy Pain Methodology Review.pdf]

Appendix – Tendinopathy and pain assessment methodology review

Electronic database search strings

PubMED

(central nervous system sensitization[MeSH] OR nociplastic*[tw] OR hyperalgesia[MeSH] OR pain threshold*[MeSH] OR sensitization[tw] OR sensitisation[tw] OR hyperalgesia[tw] OR hypersensitivity[tw] OR algometry[tw] OR vibratory detection[tw] OR vibration disappearance threshold*[tw] OR thermal detection[tw] OR quantitative sensory testing[tw] OR hyperexcitability[tw] OR wind-up[tw] OR wind up[tw] OR postsynaptic potential summation[MeSH] OR temporal summation[tw] OR neural inhibition[tw] OR altered pain threshold*[tw] OR central pain physiopathology[tw] OR nociception [tw] OR pain modulation[tw] OR pain processing[tw] OR neuropathic pain[tw] OR allodynia[tw] OR somatosensory profile*[tw] OR pain pressure threshold*[tw]) AND (athletic injuries[MeSH] OR tendinopathy[MeSH] OR tendinopathies[tw] OR overuse injuries[tw] OR jumpers knee[tw] OR jumper's knee[tw] OR patellar tendin*[tw] OR patellar tendon[tw] OR Achilles tendin*[tw] OR Achilles tendon[MeSH] OR GTPS[tw] OR greater trochanteric pain syndrome[tw] OR gluteal tendin*[tw] OR gluteal tendon[tw] OR hamstring tendons[MeSH] OR hamstring tendin*[tw] OR adductor tendon[tw] OR adductor tendin*[tw] OR posterior tibial*[tw] OR tibial tendin*[tw] OR posterior tibial tendon dysfunction[MeSH])

Scopus

(TITLE-ABS-KEY ("central nervous system") OR TITLE-ABS-KEY ("central nervous system sensitization") OR TITLE-ABS-KEY ("central sensitization") OR TITLE-ABS-KEY (nociplastic*) OR TITLE-ABS-KEY (hyperalgesia) OR TITLE-ABS-KEY ("pain threshold*") OR TITLE-ABS-KEY ("quantitative sensory testing") OR TITLE-ABS-KEY (sensitization) OR TITLE-ABS-KEY (sensitisation) OR TITLE-ABS-KEY (algometry) OR TITLE-ABS-KEY (hypersensitivity) OR TITLE-ABS-KEY (hyperexcitability) OR TITLE-ABS-KEY (nociception) OR TITLE-ABS-KEY ("pain modulation") OR TITLE-ABS-KEY ("conditioned pain modulation") OR TITLE-ABS-KEY ("temporal summation") OR TITLE-ABS-KEY ("thermal detection") OR TITLE-ABS-KEY ("wind-up") OR TITLE-ABS-KEY ("wind up") OR TITLE-ABS-KEY ("pain processing") OR TITLE-ABS-KEY (allodynia) OR TITLE-ABS-KEY ("pain pressure threshold*") OR TITLE-ABS-KEY ("pressure pain threshold*") OR TITLE-ABS-KEY ("neuropathic pain") OR TITLE-ABS-KEY ("vibratory detection") OR TITLE-ABS-KEY ("vibration disappearance threshold*") OR TITLE-ABS-KEY ("somatosensory profile*")) AND (TITLE-ABS-KEY ("patellar tendin*")) OR (TITLE-ABS-KEY ("patellar tendon") OR TITLE-ABS-KEY ("Achilles tendon") OR TITLE-ABS-KEY ("Achilles tendin*") OR TITLE-ABS-KEY ("jumpers knee") OR TITLE-ABS-KEY ("jumper's knee") OR TITLE-ABS-KEY (overuse) OR TITLE-ABS-KEY ("greater trochanteric pain

syndrome") OR TITLE-ABS-KEY (gtps) OR TITLE-ABS-KEY ("gluteal tendon") OR TITLE-ABS-KEY ("gluteal tendin*") OR TITLE-ABS-KEY ("adductor tendon") OR TITLE-ABS-KEY ("adductor tendin*") OR TITLE-ABS-KEY ("hamstring tendon") OR TITLE-ABS-KEY ("hamstring tendin*") OR TITLE-ABS-KEY ("tibial tendon") OR TITLE-ABS-KEY ("tibial tendin*")) AND (LIMIT-TO (LANGUAGE , "English"))

CINAHL

((SU (central nervous system) OR TI (central nervous system) OR AB (central nervous system)) OR (SU (central sensitization) OR TI (central sensitization) OR AB (central sensitization)) OR (SU (nociplastic*) OR TI (nociplastic*) OR AB (nociplastic*)) OR (SU (hyperalgesia) OR TI (hyperalgesia) OR AB (hyperalgesia)) OR (SU (pain threshold*) OR TI (pain threshold*) OR AB (pain threshold*)) OR (SU (quantitative sensory testing) OR TI (quantitative sensory testing) OR AB (quantitative sensory testing)) OR (SU (temporal summation) OR TI (temporal summation) OR AB (temporal summation)) OR (SU (wind up) OR TI (wind up) OR AB (wind up)) OR (SU (sensitization) OR TI (sensitization) OR AB (sensitization)) OR (SU (sensitisation) OR TI (sensitisation) OR AB (sensitisation)) OR (SU (algometry) OR TI (algometry) OR AB (algometry)) OR (SU (hyperexcitability) OR TI (hyperexcitability) OR AB (hyperexcitability)) OR (SU (hypersensitivity) OR TI (hypersensitivity) OR AB (hypersensitivity)) OR (SU (nociception) OR TI (nociception) OR AB (nociception)) OR (SU (pain modulation) OR TI (pain modulation) AB (pain modulation)) OR (TI (pain processing) OR SU (pain processing) OR AB (pain processing)) OR (TI (pain pressure threshold*) OR SU (pain pressure threshold*) OR AB (pain pressure threshold*)) OR (SU (neuropathic pain) OR TI (neuropathic pain) OR AB (neuropathic pain)) OR (SU (vibratory detection) OR TI (vibratory detection) OR AB (vibratory detection)) OR (SU (vibration disappearance threshold*) OR TI (vibration disappearance threshold*) OR AB (vibration disappearance threshold*)) OR (SU (somatosensory profile*) OR TI (somatosensory profile*) OR AB (somatosensory profile*)) OR (SU (thermal detection) OR TI (thermal detection) OR AB (thermal detection)) AND (SU (patellar tendin*) OR TI (patellar tendin*) OR AB (patellar tendin*)) OR (SU (patellar tendon) OR TI (patellar tendon) OR AB (patellar tendon)) OR (SU (Achilles tendon) OR TI (Achilles tendon) OR AB (Achilles tendon)) OR (SU (Achilles tendin*) OR TI (Achilles tendin*) OR AB (Achilles tendin*)) OR (SU (jumpers knee) OR TI (jumpers knee) OR AB (jumpers knee)) OR (SU (jumper's knee) OR TI (jumper's knee) OR AB (jumper's knee) OR (TI (overuse) OR AB (overuse) OR SU (overuse)) OR TI (greater trochanteric pain syndrome) OR AB (greater trochanteric pain syndrome) OR SU (greater trochanteric pain syndrome)) OR (TI (gtps) OR AB (gtps) OR SU (gtps)) OR (TI (gluteal tendon) OR AB (gluteal tendon) OR SU (gluteal tendon)) OR (SU (gluteal tendin*) OR TI (gluteal tendin*) OR AB (gluteal tendin*)) OR (TI (hamstring tendon) OR AB (hamstring tendon) OR SU (hamstring tendon)) OR (SU (hamstring tendin*) OR TI (hamstring tendin*) OR AB (hamstring tendin*)) OR (TI (tibial* tendon) OR AB (tibial* tendon) OR SU (tibial* tendon)) OR (SU (tibial* tendin*) OR TI (tibial* tendin*) OR AB (tibial* tendin*)) OR (TI (adductor tendon) OR AB (adductor tendon) OR SU (adductor tendon)) OR (SU (adductor tendin*) OR TI (adductor tendin*) OR AB (adductor tendin*)))

Cochrane Central Register of Controlled Trials

((“central nervous system”) OR (“central nervous system sensitization”) OR (“central sensitization”) OR (nociplastic*) OR (hyperalgesia) OR (sensitization) OR (hyperexcitability) OR (hypersensitivity) OR (nociception) OR (“temporal summation”) OR (“wind-up”) OR (“wind up”) OR (“quantitative sensory testing”) OR (algometry) OR (“vibratory detection”) OR (“thermal detection”) OR (“vibration disappearance threshold*”) OR (“pain pressure threshold*”) OR (“pressure pain threshold*”) OR (“pain processesing”) OR (“pain modulation”) OR (allodynia) OR (“neuropathic pain”) OR (“somatosensory profile*”)) AND ((“patellar tendin*”) OR (“patellar tendon”) OR (“jumper’s knee”) OR (“jumpers knee”) OR (“Achilles tendin*”) OR (“Achilles tendon”) OR (“greater trochanteric pain syndrome”) OR (gtps) OR (“gluteal tendon”) OR (“gluteal tendin*”) OR (“hamstring tendon”) OR (“hamstring tendin*”) OR (”adductor tendon”) OR (”adductor tendin*”) OR (”tibial tendon”) OR (”tibial tendin*”) OR (“overuse”))

SPORTDiscus

(((SU (central nervous system) OR TI (central nervous system) OR AB (central nervous system)) OR (SU (central sensitization) OR TI (central sensitization) OR AB (central sensitization)) OR (SU (nociplastic*) OR TI (nociplastic*) OR AB (nociplastic*)) OR (SU (hyperalgesia) OR TI (hyperalgesia) OR AB (hyperalgesia)) OR (SU (pain threshold*) OR TI (pain threshold*) OR AB (pain threshold*)) OR (SU (quantitative sensory testing) OR TI (quantitative sensory testing) OR AB (quantitative sensory testing)) OR (SU (sensitization) OR TI (sensitization) OR AB (sensitization)) OR (SU (sensitisation) OR TI (sensitisation) OR AB (sensitisation)) OR (SU (algometry) OR TI (algometry) OR AB (algometry)) OR (SU (hyperexcitability) OR TI (hyperexcitability) OR AB (hyperexcitability)) OR (SU (hypersensitivity) OR TI (hypersensitivity) OR AB (hypersensitivity)) OR (SU (nociception) OR TI (nociception) OR AB (nociception)) OR (SU (pain modulation) OR TI (pain modulation) AB (pain modulation)) OR (TI (pain processing) OR SU (pain processing) OR AB (pain processing)) OR (TI (pain pressure threshold*) OR SU (pain pressure threshold*) OR AB (pain pressure threshold*)) OR (SU (neuropathic pain) OR TI (neuropathic pain) OR AB (neuropathic pain)) OR (SU (vibratory detection) OR TI (vibratory detection) OR AB (vibratory detection)) OR (SU (vibration disappearance threshold*) OR TI (vibration disappearance threshold*) OR AB (vibration disappearance threshold*)) OR (SU (somatosensory profile*) OR TI (somatosensory profile*) OR AB (somatosensory profile*)) AND (SU (patellar tendin*) OR TI (patellar tendin*) OR AB (patellar tendin*)) OR (SU (patellar tendon) OR TI (patellar tendon) OR AB (patellar tendon)) OR (SU (Achilles tendon) OR TI (Achilles tendon) OR AB (Achilles tendon)) OR (SU (Achilles tendin*) OR TI (Achilles tendin*) OR AB (Achilles tendin*)) OR (SU (jumpers knee) OR TI (jumpers knee) OR AB (jumpers knee)) OR (SU (jumper's knee) OR TI (jumper's knee) OR AB (jumper's knee) OR (TI (overuse) OR AB (overuse) OR SU (overuse)) OR TI (greater trochanteric pain syndrome) OR AB (greater trochanteric pain syndrome) OR SU (greater trochanteric pain syndrome)) OR (TI (gtps) OR AB (gtps) OR SU (gtps)) OR (TI (gluteal tendon) OR AB (gluteal tendon) OR SU (gluteal tendon)) OR (SU (gluteal tendin*) OR TI (gluteal tendin*) OR AB (gluteal tendin*)))
